# Ancient Schwannoma of the Infratemporal Fossa: A Case Report

**DOI:** 10.22038/ijorl.2020.42914.2400

**Published:** 2020-11

**Authors:** Swagatika Samal, Anindya Nayak, Pradeep Pradhan

**Affiliations:** 1 *Department of Pathology and laboratory Medicine, All India Institute of Medical Sciences, Bhubaneswar, Odisha, India.*; 2 *Department of Otorhinolaryngology, All India Institute of Medical Sciences, Bhubaneswar, Odisha, India.*

**Keywords:** Ancient Schwannoma, Infratemporal fossa, Transparotid Transmandibular Approach

## Abstract

**Introduction:**

Ancient schwannoma of infratemporal fossa arising from the trigeminal nerve is very rare in clinical practice.

**Case Report:**

A 65-year old male presented to the outpatient department with a progressive swelling over the left parotid for 5 years and pain during chewing for 6 months which was diagnosed as benign spindle cell tumour on cytology. The tumor was excised with a combined transparotid and transmandibular cervical approach and the final pathology was confirmed to an Ancient Schwannoma.

**Conclusion:**

A giant infratemporal fossa Schwannoma extending to the parapharyngeal space masquerading as a parotid swelling is very unusual. Transparotid transmandibular excision of the infratemporal fossa tumor is an effective approach ensuring complete removal of the tumor with minimal postoperative complications and acceptable cosmoses.

## Introduction

Schwannomas are the capsulated benign neoplasm of the peripheral nerves derived from the Schwann cells (1). It was first described by Verocay in 1910. Approximately 37–45 % of schwannomas are found to involve the head and neck region and they usually arise from the nerve roots of the lower cranial nerves in the parapharyngeal space (2,3). In contrast, schwannoma in the infratemporal fossa (ITF)is very rare (4,5) and mostly arises from the trigeminal nerve, which accounts for about 0.2 % of all intracranial tumors and about 2–3 % of all intracranial schwannomas (3). Over time, degenerative changes can occur in a tumor, which could be either cystic or fatty degeneration with deposition of the hyaline material, a thick capsule, and infiltration of the histiocytes accumulation. These histological features are specifically demarcated in a long-standing schwannoma and are suggestive of an ancient schwannoma (6). The term ancient schwannoma was first coined by Ackerman and Taylor (6). Here we present a case of 65 year old male, diagnosed as IFT ancient schwannoma, where surgical excision was performed with the combined transparotid and transmandibular cervical approach.

## Case Report

**Fig 1:**
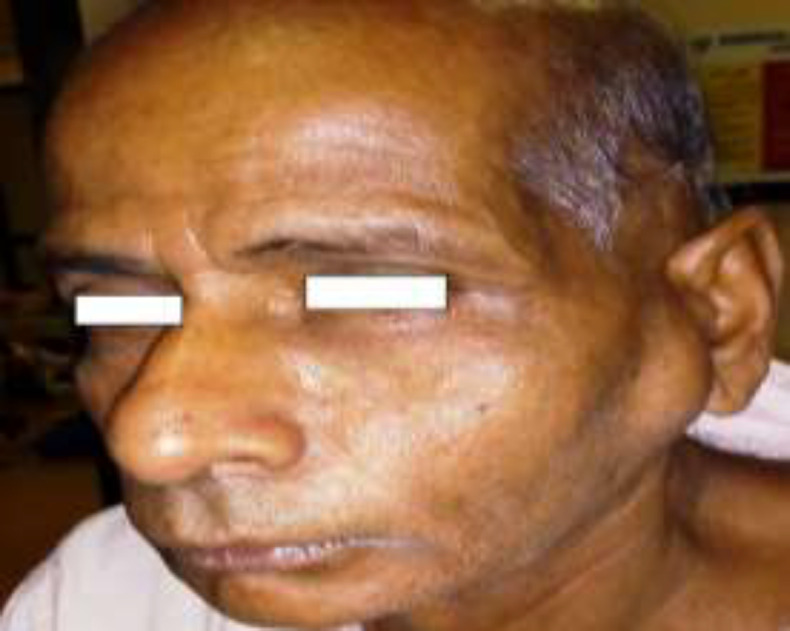
Shows a smooth parotid swelling (left) of approximately 6 cm × 5 cm size

A 65-year male presented with progressive swelling over the left parotid for 5 years and pain during chewing for 6 months. On initial examination, a smooth parotid swelling of approximately 6 cm × 5 cm was detected on the left side, which was non-tender, firms, and was partially fixed to the underlying structure (Fig 1). 

**Fig 2:**
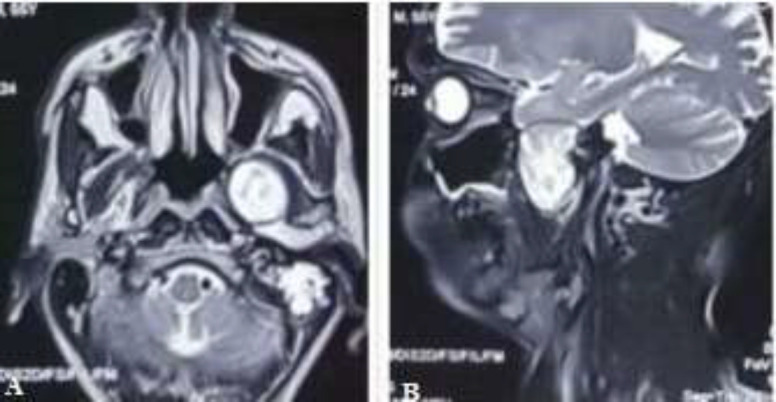
Contrast-enhanced MRI (T2- weighted) A; axial section revealed a heterogeneously enhancing mass in the infratemporal fossa, B; parasagittal section shows the extension of the tumor to the skull base

Examination of the oral cavity and oropharynx were found to be normal. There was no cervical lymphadenopathy detected in the patient. Flexible fiberoptic nasopharyngoscopy was found normal. Contrast-enhanced MRI (T2- weighted) revealed a heterogeneously enhancing mass in the left infratemporal fossa of approximately 6.4× 4.4×3.3 cm extending superiorly to the skull base and posteriorly to the parapharyngeal space with the displacement of the deep lobe of the parotid and there was no bony erosion of the skull base (Fig 2). 

**Fig 3:**
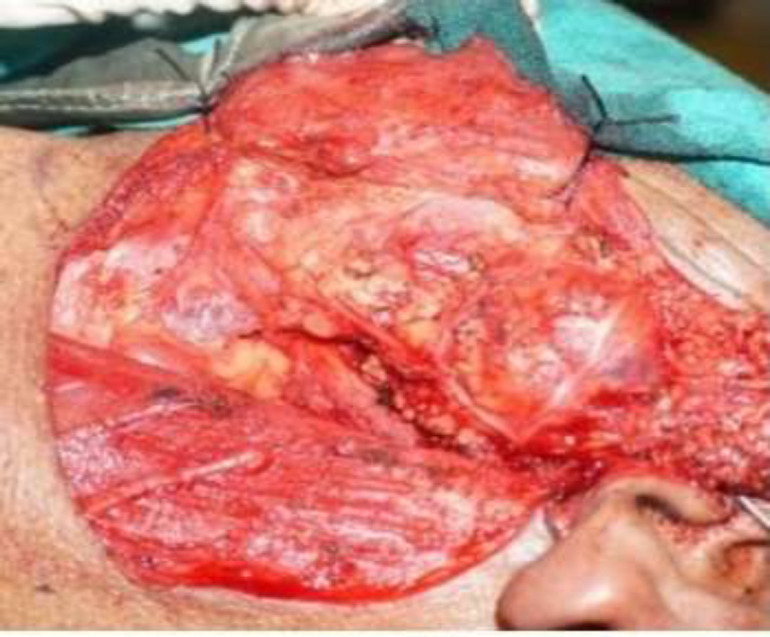
Shows the tumour bulge tumour after the superficial parotidectomy

**Fig 4:**
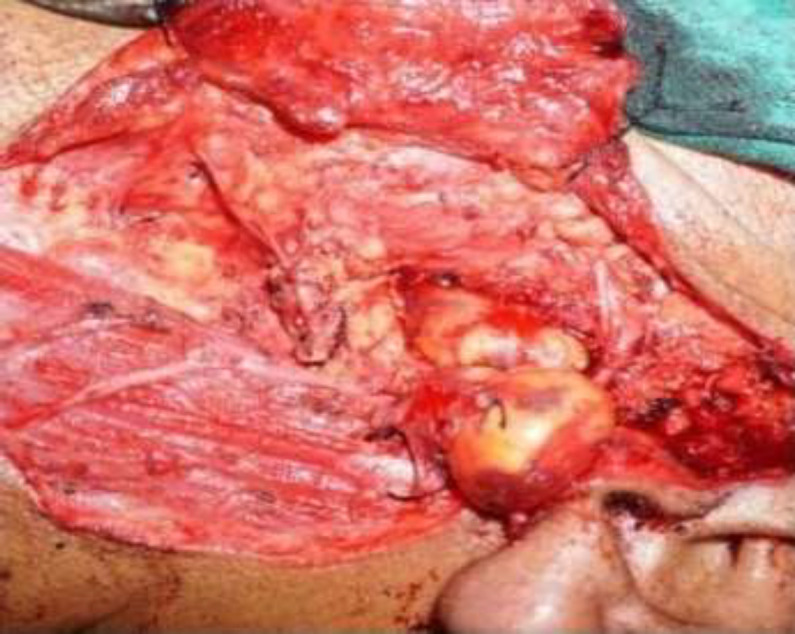
Tumour was traced anteriorly towards the infratemporal fossa

**Fig 5:**
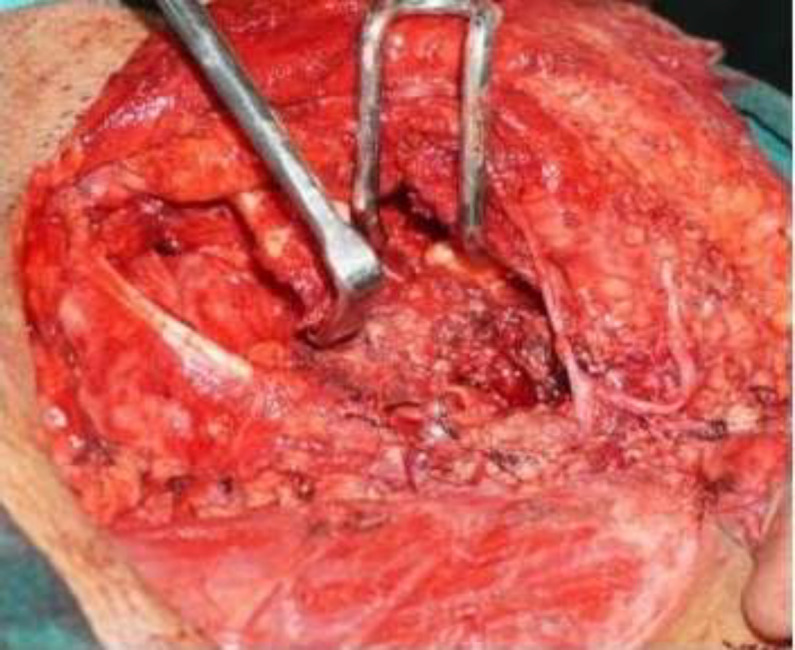
Shows the infratemporal fossa after complete excision of the tumour

All routine hematological and biochemical investigations were found normal. The fine needle aspiration cytology (FNAC) of the left parotid was performed and the findings were consistent with a benign spindle cell tumor, most probable neurogenic origin. Keeping in mind a benign neurogenic tumor,the patient consented for excision of the tumor through transparotid transmandibular approach under general anaesthesia (Fig 3, Fig 4, Fig 5). 

A preauricular incision was given from the lower border of the left zygomatic bone, extended to the neck over the skin crease. 

A total conservative parotidectomy was performed with complete preservation of the facial nerve and later, it was translocated for adequate exposure of the tumor. The carotid sheath was exposed and controlled ligature was taken over the great vessels to prevent inadvertent vascular injury during the surgery. Tumour was first identified in the parapharyngeal space, which was traced anteriorly in the infratemporal fossa. An osteotomy was done over the ascending ramus of the mandible and later retracted upwards for complete exposure of the tumor in the infratemporal fossa. Tumour was removed after a gentle dissection from the surrounding soft tissue. Ramus of the mandible was reconstructed with the titanium plates and screws. The patient had grade III facial palsy in the immediate postoperative period, which was recovered completely after two weeks of conservative treatment. 

**Fig 6:**
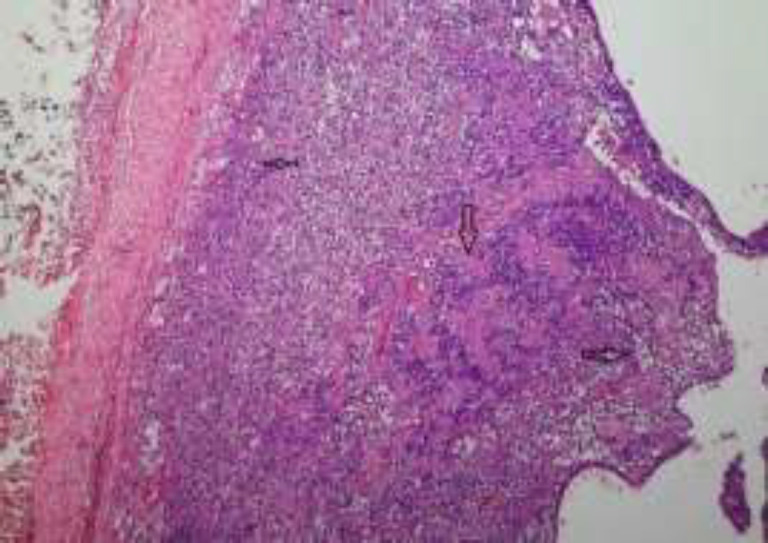
The histopathological picture revealed a well-encapsulated benign nerve sheath tumor with areas of cystic degeneration (Right black arrow). There is extensive foam cell degeneration with the presence of scattered cells having high Nucleo-cytoplasmic ratio and irregular contour (regenerative atypia, left black arrow). The middle vertical arrow indicates the microscopic pictures of nuclear palisading around an acellular eosinophilic matrix-forming verocay body, which was suggestive of schwannoma (H& E 10x)

The final histopathological report revealed a well-encapsulated tumor with areas of cystic degeneration, hyalinization, and areas of hemorrhages. The microscopic picture revealed the typical cellular arrangement, i.e., nuclear palisading around an acellular eosinophilic matrix (Antony A and Antony B) forming verocay body, which was suggestive of an ancient schwannoma (Fig 6). The patient has been on regular follow-up for the past six months and found asymptomatic without the recurrence of the disease.

## Discussion

The infratemporal fossa is a three-dimensional space bounded medially by the maxilla and the pterygoid plates, laterally by the temporalis muscles and the ramus of the mandible, and superiorly by the greater wing of the sphenoid and later forms the floor of the middle cranial fossa. It contains the medial and lateral pterygoid muscles, mandibular nerve and its branches, first and second part of maxillary vessels, otic ganglia, Vidian nerve, and sphenomandibular ligament (7). Medially it is communicated to the pterygo-palatine fossa through pterygomaxillary fissure and posteriorly to the parapharyngeal space. As trigeminal nerve predominantly occupies the ITF, schwannoma is not very uncommon in this subsite (8,9). It is a solitary, encapsulated benign neoplasm of the peripheral nerves derived from the Schwann cells. Approximately 37–45 % of them are found in the head and neck region. It is commonly found in the 2nd -3rd decade of life without having any sex predominance (7). It usually presents as a submucosal swelling, which is predominantly found in females (10). Looking into the literature, only a handful of cases of ancient schwannoma have been demonstrated and most of them were detected in relation to the oral cavity (11). Although schwannoma in the ITF is not very uncommon, documentation of an ancient schwannoma is very rare and only a few clinical cases have been published in the literature (11,12). The commonest site of origin of the ancient schwannoma in the ITF is the trigeminal nerve, mostly from the maxillary division. The primary tumor is thought to arise first in the infratemporal fossa and then it extends to the neighboring spaces (13) as, in the present case, it had involved the parapharyngeal space in producing a parotid swelling. Although pain is the frequent complaint of an ITF tumor concerned with the site of the nerve, patients can have a wide spectrum of presentations according to the extent of the lesion in the infratemporal fossa. In the present case, pain and parotid swelling were the only complaints, and there was no significant abnormality detected in the oral cavity and oropharynx examination. Radiological examination of the ancient schwannoma reveals a well-defined heterogeneous lesion due to the presence of cystic spaces, unlike the schwannoma (14). Histopathology is always the mainstay of diagnosis of the disease, and accordingly, it has been classified into 7 subtypes: classical (Verocay), cellular, plexiform, cranial nerve, melanotic, degenerated (ancient), and granular cell schwannoma (15). The histopathological picture revealed a well-encapsulated benign nerve sheath tumor with areas of cystic degeneration. There was extensive foam cell degeneration with the presence of scattered cells having a high Nucleo-cytoplasmic ratio. The microscopic picture indicates the presence of nuclear palisading around an acellular eosinophilic matrix-forming verocay body, which was suggestive of ancient schwannoma (H& E 10x) (16). All the benign tumors of parapharyngeal/infratemporal fossa, such as fibroma, lipoma, neurofibroma, or salivary gland tumor, should be excluded because of the similar clinical and radiological findings (17). As described by Dahl (18), histopathological findings may include the features of hyperchromatism and mitotic figures with insignificant verocay bodies sometimes misinterpret to a malignant tumor. Hence utmost attention should be given by the pathologist during the interpretation of the report in relation to the clinical and radiological findings. Based upon the clinical and radiological findings, the patient was suspected to be a parotid tumor extending to the infratemporal fossa, although fine needle aspiration cytology was suggestive of a spindle cell tumor. Pericapsular excision of the tumor is considered as the treatment of choice in all cases of schwannoma (19). The surgical approach depends upon the size of the tumor and its extension of into the adjacent spaces. The anterolateral route consists of frontotemporal orbitozygomatic and transcranial subtemporal frontotemporal approach. Although it provides adequate exposure to the infratemporal fossa, it is an invasive procedure with the definitive morbidity attributed to the craniotomy. Anteriorly infratemporal fossa can be assessed by the transmaxillary using a Weber Fergusson or Caldwell Luc approach and patients can have a facial scar. Nowadays, with the advancement of endoscopic sinus surgery, minimally invasive surgery has claimed to have a complete excision of the ITF tumors lesions through the transnasal and transmaxillary approach, especially for limited tumor size (20). In the present case, the tumor was extensive, involving the ITF and neighboring parapharyngeal and to the skull base. 

Keeping in mind the extension of the tumour, the patient was planned for the transparotid and transmandibular approach. Despite being a lengthy procedure and a high chance of facial nerve injury, it later provides good surgical exposure to the ITF, ensuring complete excision of the tumor. Since the preauricular incision was placed over the skin crease of the neck, the approach is well accepted by the patients because of better cosmoses. In the present case, except facial paresis, no significant postoperative complications were detected in the patient. Being a benign tumor, the prognosis is good and the malignant transformation rate is very minimal (8%-13.9%) as described in the literature (8). 

## Conclusion

Ancient schwannoma of infratemporal fossa arising from the trigeminal nerve is very rare in clinical practice. A giant infratemporal fossa schwannoma extending to the parapharyngeal space masquerading as a parotid swelling is very unusual. 

A careful correlation of the clinical and radiological findings with the histopathology is always required for the final interpretation of the biopsy report as the features of ancient schwannoma may sometimes misinterpret a malignant tumor to the surgeon and the pathologist. Transparotid transmandibular excision of the ITF tumor is an effective approach ensuring complete removal of the tumor with minimal postoperative complication and acceptable cosmoses.
